# Characteristics of diffusion-weighted and blood oxygen level-dependent magnetic resonance imaging in Tubulointerstitial nephritis: an initial experience

**DOI:** 10.1186/s12882-021-02435-6

**Published:** 2021-06-29

**Authors:** Tao Su, Xuedong Yang, Rui Wang, Li Yang, Xiaoying Wang

**Affiliations:** 1 Renal Division, Department of Medicine, Peking University First Hospital, Peking University Institute of Nephrology, Beijing, China; 2 Renal Pathology Center, Peking University First Hospital, Peking University Institute of Nephrology, Beijing, China; 3 Department of Radiology, Peking University First Hospital, Beijing, China; 4 Department of Radiology, Guang’anmen Hospital, China Academy of Chinese Medical Sciences, Beijing, China

**Keywords:** Magnetic resonance imaging, Diffusion-weighted, Blood oxygenation level-dependent, Tubulointerstitial nephritis, Acute kidney injury, Chronic kidney disease

## Abstract

**Background:**

Diffusion-weighted (DW) and blood oxygen level-dependent (BOLD) magnetic resonance imaging are classical sequences of functional MR, but the exploration in non-transplanted kidney disease is limited. Objects: To analyze the characteristics of apparent diffusion coefficient (ADC) and R_2_* value using DW and BOLD imaging in tubulointerstitial nephritis (TIN).

**Methods:**

Four acute TIN, thirteen chronic TIN patients, and four controls were enrolled. We used multiple gradient-echo sequences to acquire 12 T2*-weighted images to calculate the R_2_* map. DW imaging acquired ADC values by combining a single-shot spin-echo echo-planar imaging pulse sequence and the additional motion probing gradient pulses along the x,y, z-axes with two *b* values:0 and 200, as well as 0 and 800 s/mm^2^. ATIN patients performed DW and BOLD magnetic resonance at renal biopsy(T_0_) and the third month(T_3_). We assessed the pathological changes semiquantitatively, and conducted correlation analyses within functional MR, pathological and clinical indexes.

**Results:**

In ATIN, ADCs were significantly lower(*b* was 0,200 s/mm^2^, 2.86 ± 0.19 vs. 3.39 ± 0.11, *b* was 0,800 s/mm^2^, 1.76 ± 0.12 vs. 2.16 ± 0.08, *P* < 0.05) than controls, showing an obvious remission at T_3_. Cortical and medullary R_2_* values (CR_2_*,MR_2_*) were decreased, significant difference was only observed in MR_2_*(T_0_ 24.3 ± 2.1vs.T_3_ 33.1 ± 4.1,*P* < 0.05). No relationship was found between functional MR and histopathological indexes.MR_2_* had a close relationship with eGFR (R = 0.682,*P* = 0.001) and serum creatinine(R = -0.502,*P* = 0.012). Patients with lower ADC when *b* was 0,200 s/mm^2^ showed more increase of ADC(R = -0.956,*P* = 0.044) and MR_2_*(R = -0.949,*P* = 0.05) after therapy. In CTIN group, lowered MR_2_* and MR_2_*/CR_2_* provided evidence of intrarenal ischemia. CTIN with advanced CKD (eGFR< 45) had significantly lower ADC_*b200*_ value.

**Conclusions:**

We observed the reduction and remission of ADC and R_2_* values in ATIN case series. ATIN patients had concurrently decreased ADC_*b800*_ and MR_2_*. The pseudo normalization of CR_2_* with persistently low MR_2_* in CTIN suggested intrarenal hypoxia.

## Background

Functional magnetic resonance (fMR) imaging has recently grown to be a useful tool to evaluate real-time renal function [1]. The functional MR sequences mainly include blood oxygen level-dependent (BOLD), diffusion-weighted (DW) imaging, arterial labeling perfusion, and dynamic contrast-enhanced imaging. They provide information about diffusion, perfusion, and oxygenation of kidneys besides morphological parameters. In acute kidney injury (AKI) and chronic kidney disease (CKD), these novel techniques have been proposed as promising markers for diagnosis and provide insight into the pathophysiology of kidney disease [2–7]. In the field of kidney transplantation, fMR is recommended as a tool for early differential diagnosis of graft dysfunction [8, 9]. Researches with fMR were exploratory in animal models and some human observational studies of AKI [10–12], and in CKD patients trying to compare fMR parameters with renal pathological index [3, 10]. By contrast, fMR imaging’s role against pathological findings on non-transplantation human kidney diseases remains poorly understood.

DW and BOLD from magnetic resonance imaging are early used techniques. The total apparent diffusion coefficient (ADC) yielded from DW MR images is influenced by pure diffusion and perfusion-dependent diffusion in the low diffusion weighting (*b-*value) range. It is regarded as a marker sensitive to alterations in the interstitium, for example, interstitial edema with inflammatory cells infiltration or fibrosis, perfusion, and water handling in the tubular compartment. DW magnetic resonance imaging (MRI) helps diagnose acute renal transplant dysfunction [13]. The ADC value declined with the severity of renal dysfunction and a degree of renal fibrosis after prerenal ischemia-induced AKI [14]. But in another earlier study by Inoue T et al. [15], they found that neither ADC values nor T2* values correlated with eGFR in AKI. One possible explanation is that they enrolled complicated pathological types of AKI, including prerenal AKI, acute interstitial nephritis, and glomerulonephritis. Also, studies of ADC values in CKD got conflicting results relating to fibrosis [11].

BOLD MRI provides an index for deoxyhemoglobin levels within a defined volume of tissue (R2*), which was demonstrated to effectively assess changes in intrarenal oxygenation by measuring the R_2_* levels of the renal cortex and medulla [10]. The evolution of R2* pre-and post-furosemide injection (renal tubular response to furosemide, evaluated by the furosemide-induced suppression of oxygen consumption, FSOC) was related to impaired renal function [16], being able to make a differentiation between kidneys with preserved tubular function and tubular dysfunction. Li et al. observed the immediate increase in R_2_* in the renal inner stripe of the outer medulla after the injection of contrast agent, suggesting that hypoperfusion probably induced renal hypoxia [17], and BOLD MRI served as a sensitive method for early detection of contrast-induced AKI. Researchers found renal medullary R_2_* was decreased after furosemide injection In CKD patients with renovascular stenosis due to reduced oxygen consumption of tubular Na-K transporter working [18]. While in another report, renal BOLD MR imaging was found not to reflect renal function in CKD [19]. Some researchers recommend direct methods such as phase-contrast MRI to measure renal artery blood flow (RBF) as a surrogate for BOLD. But previous analysis [20] failed to demonstrate associations between R_2_* and RBF or sodium absorption, the correlation to RBF was poor, especially in advanced CKD with lower GFR [21]. Therefore, BOLD MRI still has its clinical value in the detection of oxygenation and renal dysfunction.

The pathogenesis of tubulointerstitial nephritis (TIN) lies in damage of tubules, changes of inflammation, edema, or subsequent fibrosis involving corresponding interstitial regions, and following regulation of intrarenal microcirculation. At the same time, glomeruli are initially intact [22]. Besides, tubulointerstitial changes after kinds of glomerular diseases also play a crucial role in disease progression. Therefore, TIN is a perfect disease model for the preliminary study of fMR characteristics in kidney diseases with varied pathological types. The study aimed to observe BOLD and DW magnetic resonance imaging characteristics in patients with acute and chronic tubulointerstitial nephritis.

## Methods

### Patients

Our study included acute tubulointerstitial nephritis (ATIN), chronic tubulointerstitial nephritis (CTIN), and healthy control in a 1: 2: 1 ratio from Jan 2008 to Jan 2009. ATIN patients were included if they (a) were adults diagnosed with biopsy-proven ATIN, (b) were capable of undergoing fMR examination three days within percutaneous renal biopsy, (c) had no signs of other kidney diseases both clinically and pathologically. CTIN patients were selected from our specialty clinic for all-cause tubulointerstitial nephritis (TIN) diseases. Patients were included if they (a) were adults clinically diagnosed with CTIN, (b) clinically had no signs of other kidney diseases and been followed up for more than one year, (c) with a stable serum creatinine level at CKD stage 2–5 [23] and well-controlled hemoglobin level in the recent three months. Healthy volunteers were recruited if they (a) were adults with no history of renal or cardiac diseases and (b) had average serum creatinine concentrations one week before MR scanning. Patients with renal malignancy, malformation, and history of partial nephrectomy were excluded from the study. Patients were required not to use diuretics one week before fMR imaging. ATIN patients performed serum creatinine (SCr) and hemoglobulin routinely for six months (T_6_). BOLD and DW imaging was carried out at renal biopsy(T_0_) and the third month after therapy (T_3_).

This prospective study was complied with the declaration of Helsinki and approved by the Human Ethics Committee of Peking University First Hospital. All subjects provided written informed consent and were compatible with MR scanning.

### MR imaging

All patients underwent MR imaging with a 3.0-T MR scanner (General Electric Medical Systems, Milwaukee, WI, USA). A multiple gradient-echo (mGRE) sequence was used to acquire 12 T2*-weighted images to calculate the R_2_* map. The parameters of sequence were as follows: TR/TE/Flip angle/BW/matrix/ thickness/gap = 100 ms/6.7–32.1 ms(12echoes)/45^0^/31.3 kHz/128 × 96/5 mm/1 mm.NEX = 1 and five to 6 axial slices were acquired within one breath-hold 24 s. DW imaging was acquired by combining a single-shot spin-echo (SE) echo-planar imaging (EPI) pulse sequence and the additional motion probing gradient (MPG) pulses along the x, y, z-axes. The parameters were as follows: TR/TE/BW/matrix = 2300 ms/56.1 ms/250 kHz/128 × 128. NEX = 2 and the slice position were identical to the BOLD imaging by the “copy” function embedded in the MR scanner, which was scanned within 18 s. We used two different b value group:0 and 200 s/mm^2^ as well as 0 and 800 s/mm^2^. We acquired axial images for both BOLD and DW images.

**Fig. 1:**
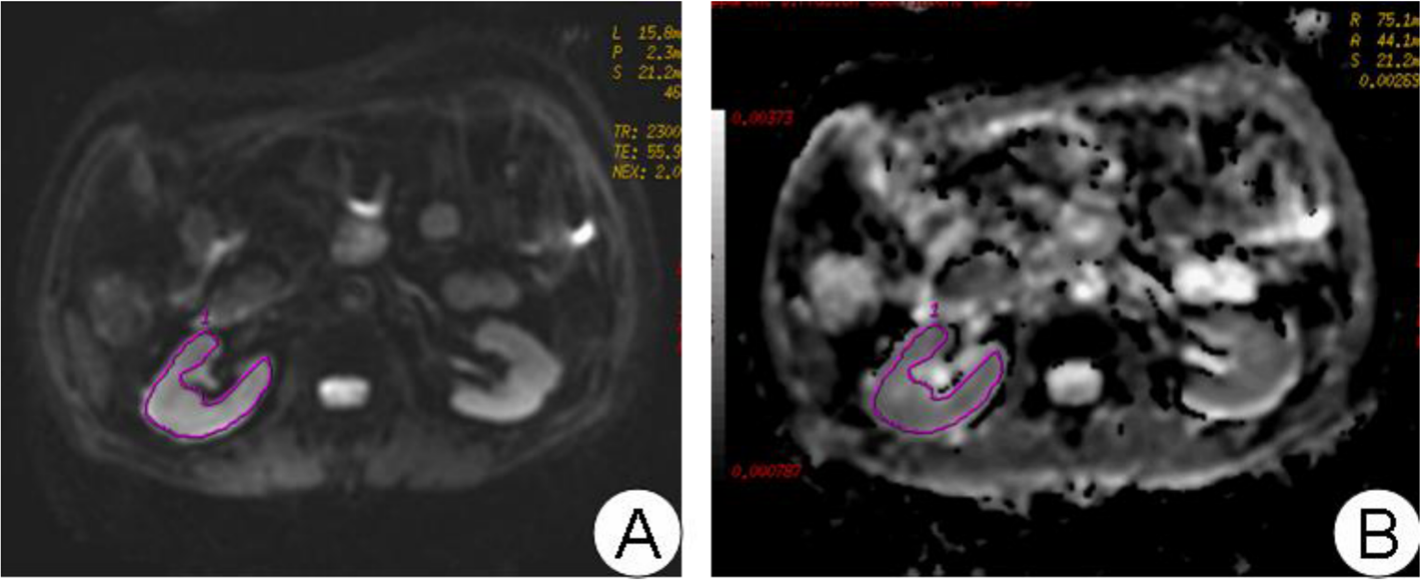
**A**: DWI image. **B**: The corresponding ADC diagram. The method of manual placement of ROI is used to outline the kidney on the anatomical map with DWI

**Fig. 2:**
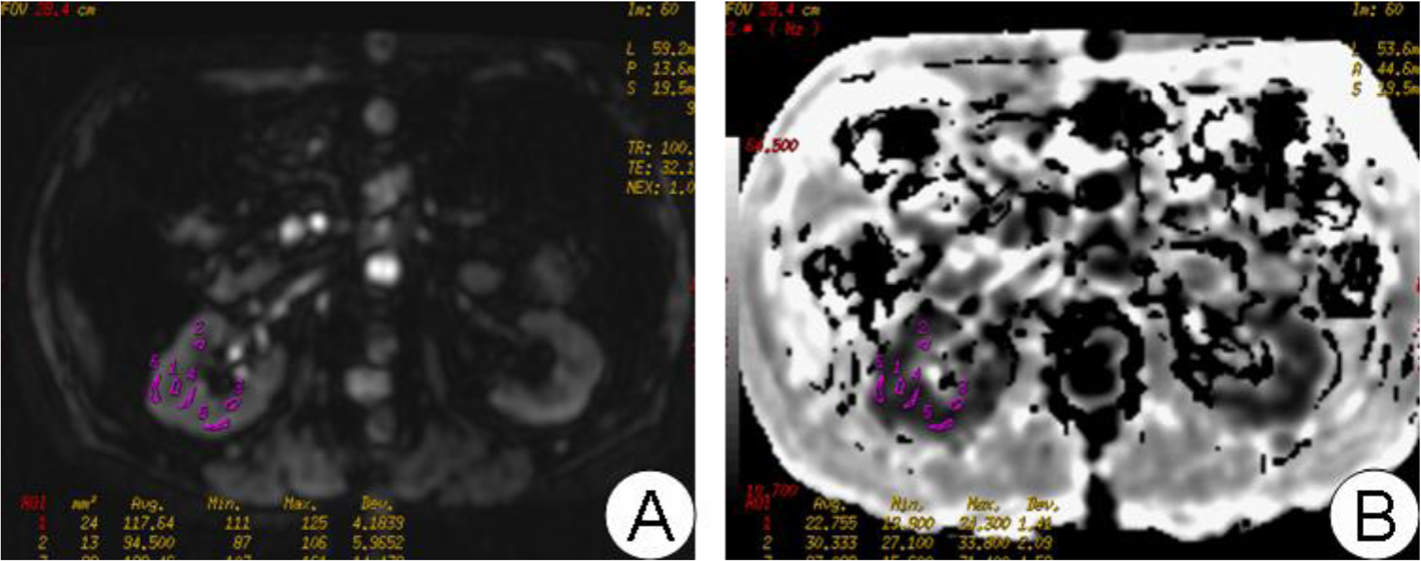
**A**: BOLD image anatomical template. **B**: The corresponding R2* figure. The manually placing ROI on the anatomical template is to place at least three ROI in the cortical medulla and read the corresponding R2* value on the corresponding R2* diagram

Both R_2_* map and apparent diffusion coefficient ADC map were generated on an AW 4.2 workstation (General Electric Medical Systems, Milwaukee, WI, USA) using “Functool” software. The reader was blinded to the subject’s clinical information. At least eight regions of interest (ROIs), each area of which was at least 10 pixels, were carefully placed on the cortex and medulla on the corresponding anatomical template separately (using image of TE = 32.1 ms as a template), the measured slices covered most of the kidney. Because of the low resolution of the images, particularly in severe renal impairment patients, it was impossible to reliably discriminate between the cortex and the medulla, which meant that the ROIs could not be reliably placed. Hence it was only possible to calculate global ADCs for each kidney. The ROIs were manually delineated in the parenchyma of the kidneys. Both R_2_* and ADC values were read out on the corresponding R_2_* and ADC map (Figs. 1-2). The cortical R_2_* (CR_2_*), medullary R_2_* (MR_2_*), and global ADC of the kidneys were calculated separately for each side.

### Pathology

Renal tissues from four ATIN patients were handled routinely by Haemotoxylin-Eosin, Masson’s trichrome, periodic acid-Schiff, and periodic acid-silver methenamine staining for light microscopy examination. The tissue core was often obtained at depths of about 1 cm. The histopathological indexes included tubular injuries (tubular epithelial cells atrophy, vacuolar degeneration, brush border shedding, necrosis, and tubulitis) and interstitial changes (edema, inflammation, and fibrosis). Area and degree of tubular brush border shedding, atrophy, and interstitial change were semiquantitatively assessed as scores 1, 2, 3, and 4 correspondings to not, mild, moderate, and severe changes by two different pathologists referring to a modification of the Banff Working Classification [24, 25]. They were also blind to the clinical data. The activity index (AI) was the total score for tubular injuries, interstitial edema, and inflammatory infiltration. The chronicity index (CI) was the total of the scores for tubular atrophy and interstitial fibrosis.

### Statistical analysis

Statistical analyses were performed using the software SPSS Version 20.0 (IBM Corp., Armonk, NY). Data were presented as the median and range. The non-parametric Kruskal-Wallis test analyzed differences between groups. Correlations were assessed according to the Pearson test for parametric data and the Spearman test for non-parametric data. The correlations between serum creatinine, eGFR, albuminuria, and the fMR parameters (kidney volume, ADC value, and R_2_* value) of all kidneys were determined. The correlations between pathological indexes and fMR parameters were also analyzed. A *P*-value less than 0.05 was defined as statistically significant.

## Results

### Clinical and pathological characteristics

**Fig. 3:**
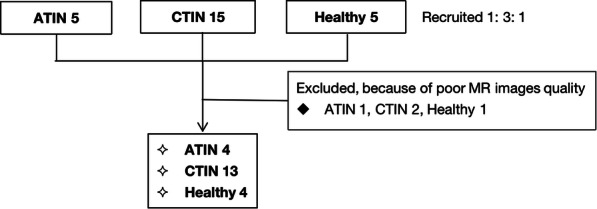
The flowchart of the study

**Fig. 4:**
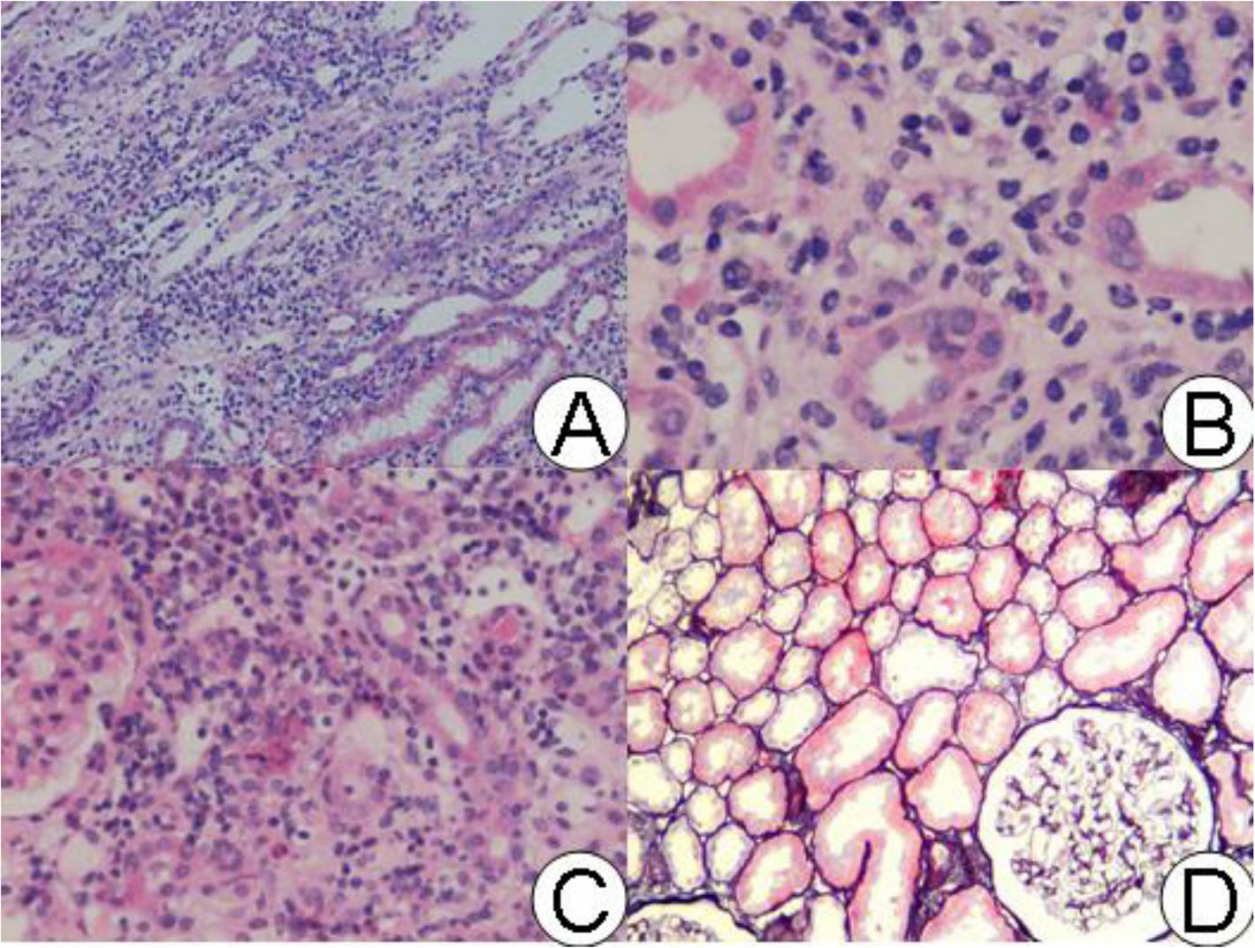
Pathological pictures of ATIN and normal kidney (all HE staining). A: example 1, magnification 100xdexB: case 2, magnification × 400 × C: case 3, magnification × 200. From A to C, ATIN showed exfoliated brush margin of renal tubules, dilated lumen, diffuse edema of the renal interstitium, multifocal or diffuse (C) lymphoid and monocytes infiltration, and eosinophils infiltration. There were no obvious pathological changes in glomeruli and arterioles. Fig. D shows normal kidney, magnification × 100: glomeruli and tubules are normal

There were 20 individuals recruited for this study, including 5 ATIN patients, 15 CTIN patients, and five healthy control. Four patients were excluded from the study because their fMR images’ quality was poor to be used (Fig. 3. flow chart). Thus, four patients with ATIN, thirteen patients with CTIN, and four healthy control were finally enrolled. The demographic and clinical data of the subjects are summarized in Table 1. The ATIN patients were 43.8 ± 19.4 years old, with an average of 52.0 ± 13.3 years old of CTIN patients. All the ATIN patients who experienced acute kidney injury (AKI) were defined using the Kidney Disease: Improving Global Outcomes (KDIGO) [26, 27] criteria and consensus report of the Acute Disease Quality Initiative 16 Workgroup. One patient was in AKI stage 1, two in AKI stage 2, and one in AKI stage 3. Renal pathology revealed that in ATIN kidneys, the glomeruli were relatively intact. Focal or diffuse tubular injuries, diffuse interstitial edema, and mononuclear cell infiltration were predominant pathological findings (Fig. 4). The activity index was averaged 12.8 ± 3.3, with the chronicity index 3.5 ± 0.6. Increased urinary albumin was at an average of 102.0 ± 65.9 mg/L. The Scr level was 112 ~ 401 μmol/l (217.4 ± 126.4 μmol/l, eGFR 37.4 ± 31.5 ml/min/1.73m^2^) at renal biopsy, and gradually declined to average level after short-term steroids administration during the following three months, the eGFR was averaged 66.6 ± 31.2 ml/min, 74.4 ± 41.1 ml/min at the third (T_3_) and sixth month (T_6_). The hemoglobulin of ATIN patients was 99.0 ± 13.4 g/L initially and corrected to 129.0 ± 13.2 g/L.

**Table 1:** Laboratory and fMR data of ATIN and CTIN kidneys

	Age	Scr (μmol/l)	eGFR (ml/min)	Hb (g/L)	CR_2_* (Hz)	MR_2_* (Hz)	CR_2_* /MR_2_*	ADC value *b* value 800 (s/mm^2^)	ADC value *b* value 200 (s/mm^2^)	*Volume* *(cm3)*
Control	50.3 ± 10.0	72.3 ± 11.5	98.0 ± 6.7	135.2 ± 5.2	19.7 ± 2.1	33.1 ± 4.1	1.68 ± 0.03	2.16 ± 0.08	3.39 ± 0.11	144.6 ± 16.8
ATIN T0	43.8 ± 19.4	217.4 ± 126.4 (112 ~ 401)	37.4 ± 31.5 (9.9 ~ 82.8)	99.0 ± 13.4	17.6 ± 1.3	24.3 ± 2.1#	1.39 ± 0.13	1.76 ± 0.12#	2.86 ± 0.19#	176.8 ± 82.8
T3		103.3 ± 15.8	66.6 ± 31.2	129.0 ± 13.2	18.3 ± 2.2	32.4 ± 6.6	1.8 ± 0.52	2.02 ± 0.04*	3.41 ± 0.10*	154.2 ± 7.0
T6		97.5 ± 21.0 (76 ~ 118)	74.4 ± 41.1 (43.6 ~ 132.2)	134.8 ± 8.6	N.D.	N.D.	N.D.	N.D.	N.D.	N.D.
CTIN	52.0 ± 13.3	240.2 ± 146.0 (102 ~ 526)	34.7 ± 21.9 (5.1 ~ 79.8)	126.8 ± 16.8	19.0 ± 2.2	28.0 ± 5.0	1.32 ± 0.69	2.20 ± 0.20	3.46 ± 0.43	89.0 ± 23.0^
eGFR< 45								2.23 ± 0.21	3.14 ± 0.30^※^	
eGFR> 45								2.15 ± 0.19	3.66 ± 0.37	

Note: CR_2_*, Cortical R_2_*; MR_2_*, Medullary R_2_*, N.D. no data

# when compared between T0 and control, the difference is significant *P* < 0.05

* when compared between T0 and T3, the difference is significant *P* < 0.05

no significant difference was found between T3 and control

※when compared with group eGFR> 45 ml/min, the difference is significant P < 0.05

^ when compared with T0, T3 and control, *P* < 0.005

Thirteen patients with CTIN were enrolled from our out-patient specialty for tubulointerstitial nephritis under integrative supportive therapy for CKD. The renal function was stable the recent three months before MR imaging. Patients were in CKD stage 2 ~ 5 non-dialysis, whose eGFRs were averaged 34.7 ± 21.9 ml/min (Scr 102 ~ 526 μmol/l). Urinary albumin was 30.0 ± 17.5 mg/L. Renal anemia of CTIN patients had already been corrected to 126.8 ± 16.8 g/L, which was matchable with healthy control.

### Functional MR imaging features

**Fig. 5:**
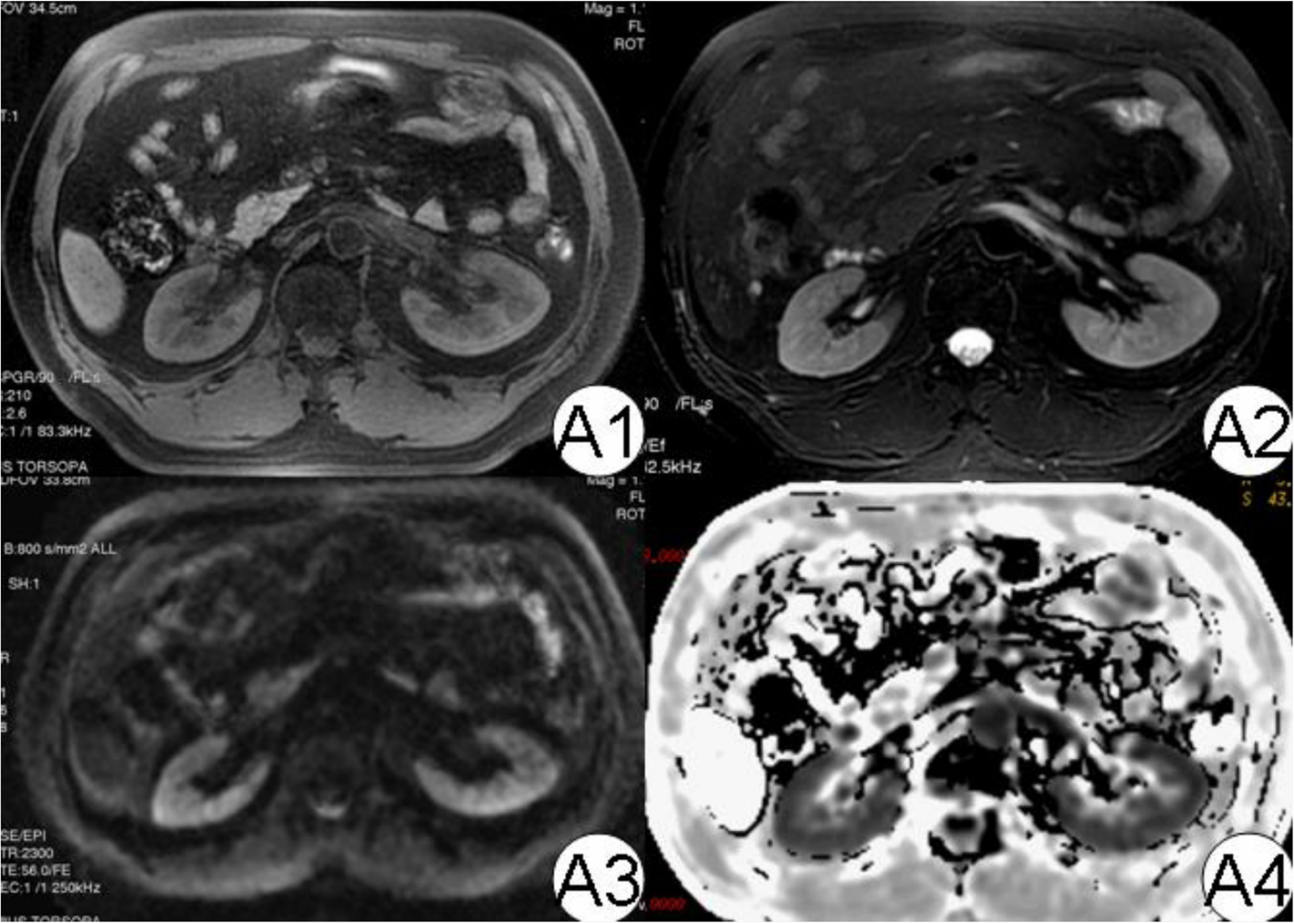
MRI diagram of normal kidney. In the picture, A1 to A4 are T1WI, T2WI, DWI, and R2*, respectively. It can be seen that the demarcation of the epithelium and medulla on T1WI, T2WI, and R2 * maps is clear (3 points)

In control kidneys (Fig. 5), the outline was smooth, and there was a clear differentiation between renal cortex and medulla on T_1_-weighted SE and IR sequence. The average volume of kidneys was (144.6 ± 16.8) × 10^3^ mm^3^. Global ADC values of DW imaging were 3.39 ± 0.11 and 2.16 ± 0.08, respectively, when *the b* value was 0, 200, or 0, 800 s/mm^2^. CR_2_* value yielded from BOLD MRI was 19.7 ± 2.1 Hz, which was lower than medulla [(33.1 ± 4.1) vs. (19.7 ± 2.1) Hz, *p* < 0.05]. Thus, the ratio of MR_2_* to CR_2_* was 1.68 ± 0.03.

**Fig. 6:**
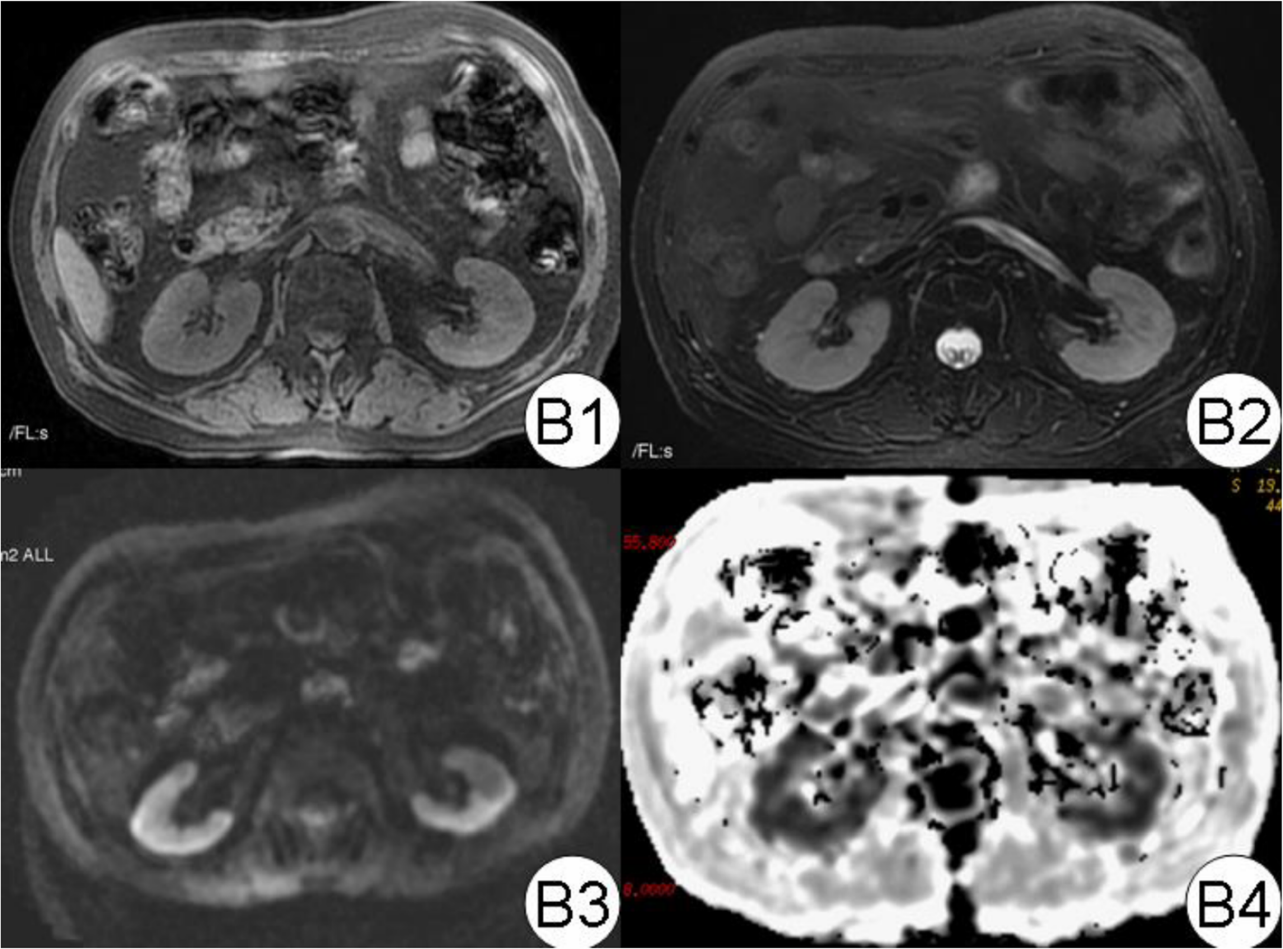
ATIN kidney MRI diagram. In the picture, B1 to B4 are T1WI and T2WI DWI and R2* pictures, respectively. It can be seen that the corticomedullary boundary between T1WI and T2WI is OK (2 points). Compared with normal, the cortical area of the R2 * map is slightly irregular

**Fig. 7:**
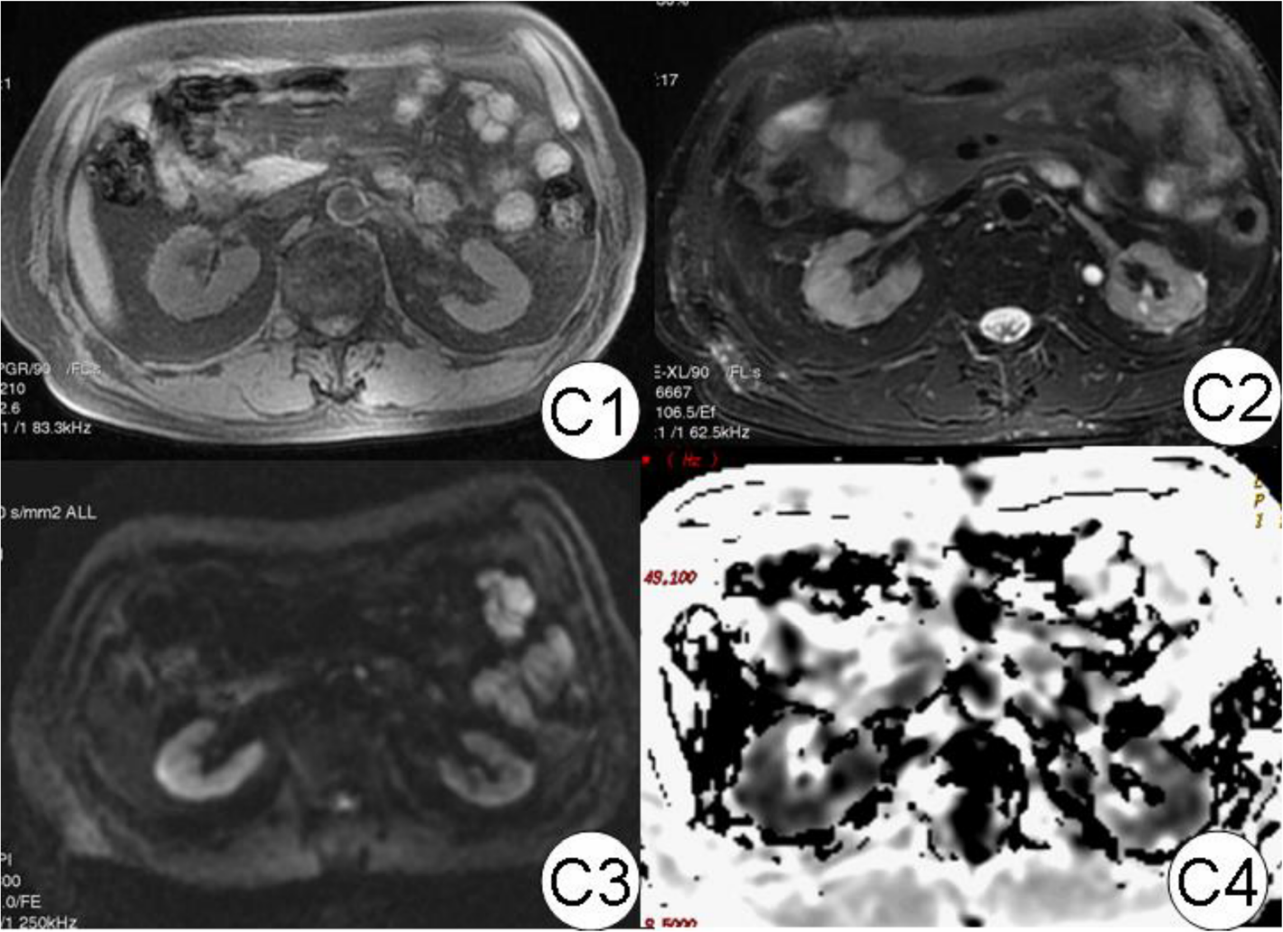
CTIN kidney MRI diagram. In the figure, C1 to C4 are T1WI and T2WIMagol DWI and R2* pictures, respectively. The epithelial medulla boundary of T1WI and T2WI could only be seen faintly (1 point). When compared with normal and ATIN, the epithelial medulla of R2 * was irregular

**Fig. 8:**
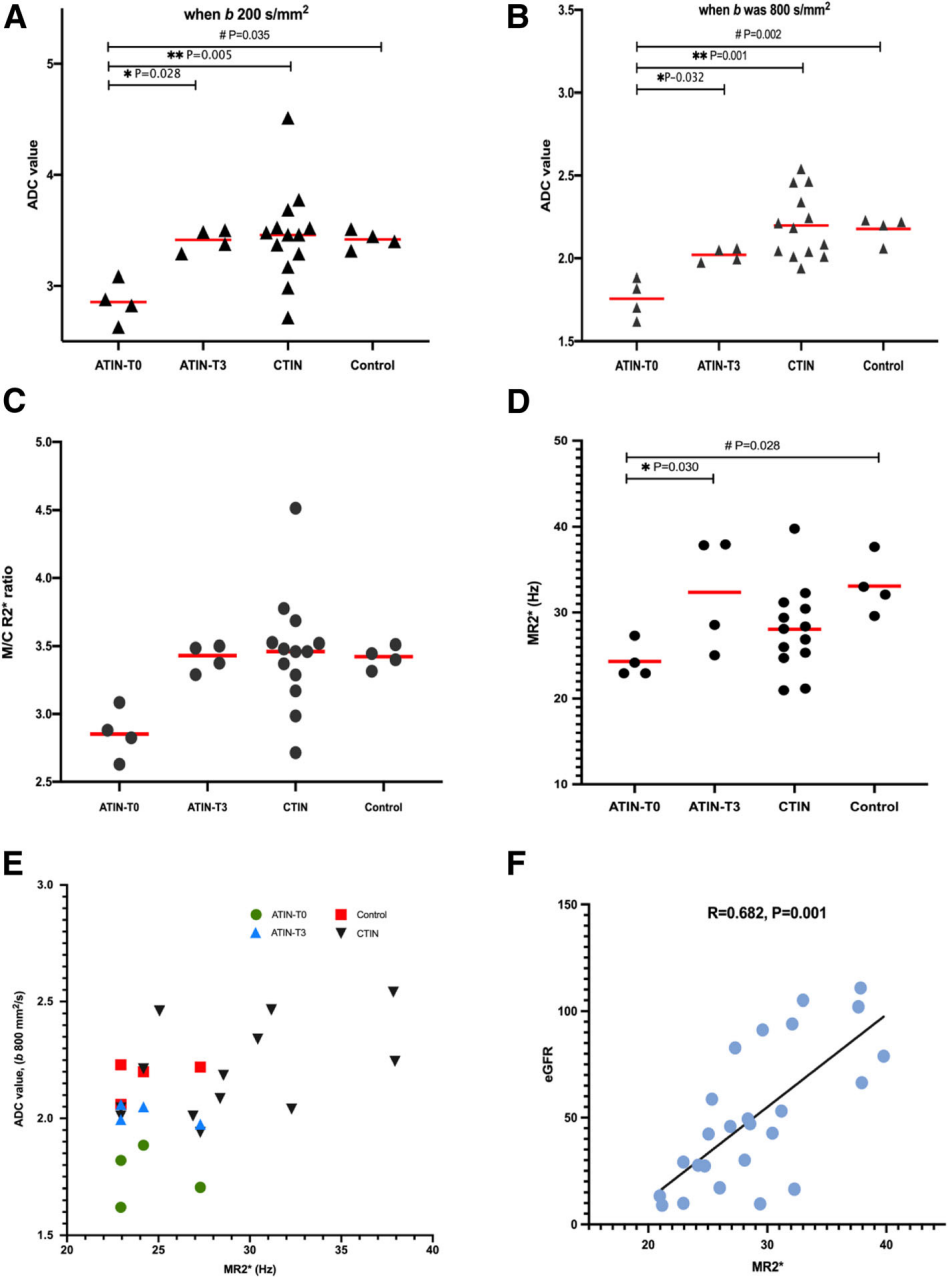
The correlation analysis of DWI, BOLD parameters and eGFR

In ATIN patients, swollen kidneys were observed (Fig. 6). The volume was (176.8 ± 82.8) × 10^3^ mm^3^. ADC values were obtained in DWI both when *b* value was 0, 200 s/mm^2^ and 0, 800 s/mm^2^, and all were found to have significant decreases as 18.5, 15.6% respectively than the control group (Table 1, Figs. 7A-8B). We observed the obvious rising of decreased ADC values following renal function improvement to achieve a 93.5, 100% recovery in the third month (see Table 1). Both CR_2_* and MR_2_* values of ATIN kidneys were lowered than controls at renal biopsy; the difference was significant in MR_2_* values as 26.6% (difference in CR_2_* values was 10.7%). The MR_2_* values firstly went back to a level similar to control, while the CR_2_* values had not yet fully recovered (92.9% of control). The MR_2_* to CR_2_* ratio (M/CR_2_*) was 1.39 ± 0.13, 1.8 ± 0.52 respectively, in the acute and remission stage. For CTIN patients (Fig. 8), extremely atrophic kidneys with irregular outlines were found (Fig. 1). The volume was 89.0 ± 23.0 × 10^3^ mm^3^. Both ADC values when *b* values 0, 200 s/mm^2^ and *b* value 0,800 s/mm^2^ were similar as healthy control. In the R_2_* map, MR_2_* values of CTIN kidneys were averaged 28.0 ± 5.0 Hz, which was lower than control, but the difference was not statistically significant. M/CR_2_* ratio was low as 1.32 ± 0.69.

Correlation analysis disclosed that neither ADC values nor R_2_* values correlated to histopathological indexes, including tubular injuries (tubular epithelial cells atrophy, vacuolar degeneration, brush border shedding, necrosis, and tubulitis) and interstitial changes (edema, inflammation, and fibrosis) when compared separately. We also found no relationship within ADC values, R_2_* values, AI and CI. It seems that ADC and R_2_* values changed along with that of renal function (Table 1) in ATIN kidneys, while a close relationship was identified only between MR_2_* values and eGFR (R = 0.8, *P* = 0.017), and Scr (R = -0.502, *P* = 0.012). The association was similar in CTIN patients (R = 0.615, *P* = 0.025) or all the TIN patients (R = 0.682, *P* = 0.001, Fig. 8F). There was also a correlation between M/CR_2_* and eGFR in the TIN (R = 0.659, P = 0.00), but differences were not significant within subgroups (Fig. 8C). ADC_*b800*_ was inversely correlated with albuminuria (R = -0.951, P = 0.001) in ATIN patients, while had no such relationship in CTIN. We noticed that significantly lowered ADC_*b200*_ values occurred in advanced CKD patients with eGFR< 45 ml/min (when compared with eGFR> 45 ml/min: 3.14 ± 0.30 vs. 3.66 ± 0.37, *P* < 0.05). We could figure out from Fig. 8E that ATIN kidneys having relatively lowest ADC_*b800*_ and MR_2_* (green dots), distinguished from other diseases. Further analysis revealed that ATIN Patients with lower ADC_*b200*_ were correlated with more *Δ*ADC_*b200*_ (the increase of ADC_*b200*_ value over the following three months, R = -0.956, *P* = 0.044) and *Δ*MR_2_* (the increase of MR_2_* value over the following three months, R = -0.949, *P* = 0.05) after therapy. There was also a relationship between *Δ*ADC_*b200*_ and *Δ*MR_2_* (R = 0.995, *P* = 0.005). However, renal prognosis analysis among candidate predictive markers showed no relationship between time-point ADC or R_2_* values and eGFR. But as MR_2_* has a significant correlation to eGFR and Scr levels, it was speculated that a lower ADC_*b200*_ value could predict the degree of ADC and MR_2_* recovery after therapy, which meant a better restoration of renal function.

## Discussion

With MR techniques and clinical applications’ developments, it is possible to make a noninvasive assessment of renal pathology types by fMR imaging in patients with kidney diseases. However, at present, there is little evidence directly from kidney histopathology [28–31]. In this study, DW and BOLD MRI parameters of TIN kidneys were acquired and compared between ATIN and CTIN. We also provided sequential changes of fMR in the remission stage of ATIN. By analyzing the correlation of fMR parameters with critical pathological and clinical factors, we explore the potential significance of these novel techniques as noninvasive methods, contributing to pathological diagnosis and long-term prognosis assessment, which would enrich our understanding of tubular interstitial diseases.

Diffusion-weighted MR imaging generates the ADC value as an index reflecting the microenvironment of diffusing water molecules. It is considered a simple marker to reflect tissue microstructure. In renal dysfunction, tubular injuries lead to a reduced water reabsorption process resulting in decreased diffusion [32]. Factors involving microcapillary perfusion, the status of tissue edema, and fibrosis also theoretically dedicate to ADC. Xu Y et al. [33] reported that ADC values of impaired kidneys were significantly lower in a linear but positive correlation with eGFR in CKD patients with renal arterial stenosis. In diabetes nephropathy, ADC values positively correlated to eGFR values, inversely to clinical stages and urinary albumin excretion [34]. But in the AKI study mentioned above [15], neither ADC nor T2* values (R2* = 1/T2*) were correlated with eGFR. One possible explanation is that they enrolled complicated pathological types of AKI, including prerenal AKI, acute interstitial nephritis, and glomerulonephritis. In the current study, there were significantly decreased on initial stage but reversible changes of ADC values in remission (both *b* values) in ATIN patients self-pre-and post-therapy. The delayed recovery of ADC, especially when *b* was 0, 800 s/mm^2^ in the remission stage, meant that kidney injury was still in repair or left behind chronicity. The decrease of ADC (*b* was 0, 800 s/mm^2^) in CTIN patients was mild, suggesting its insensitivity to fibrosis. The finding relationship of albuminuria with ADC_*b800*_ shares the same view of previous studies [34, 35]. Only ADC_*b200*_ served as a factor determined by perfusion and interstitial changes, showed better recognition between mild and severe CKD (eGFR< 45) in our CTIN patients. This result was in line with a recently published study by Buchanan CE et al. [36] that ADC differed between low/high interstitial fibrosis groups at 30–40% fibrosis threshold in CKD. Combined action from concurrent tubular injuries, interstitial inflammation aggravated fibrosis, and intrarenal hypoperfusion in the process of CTIN make the change of ADC value more uncertain. It lacks sensitivity to distinguish edema from fibrosis.

As we have known, the R_2_* value of BOLD MR imaging has been regarded as a factor reflecting tissue oxygenation [30]. Intrarenal hypoxia contributes to the deterioration of CKD [37]. The changes in cortical oxygenation are usually influenced by nephrotoxicity and severe hemodynamic instability. In a rat model of Gentamycin-induced AKI, only CR_2_* was decreased while MR_2_* remained constant [38]. This is because gentamycin has direct nephrotoxicity to proximal tubular epithelial cells. The renal medulla is more vulnerable to hypoxia than the cortex because of low oxygen delivery due to low vascular density in the medulla, arterial-venous shunting, and high oxygen consumption for active transcellular transport of sodium in the thick ascending limb of the Henle loop (the target site of inhibitors of the sodium-potassium-chloride cotransporter). A study of the renal response to furosemide showed that medulla oxygenation was increased after furosemide injection while cortical and medullary perfusion remained constant [16]. Renal hypoperfusion leads to a more significant medullary oxygenation change than that of the cortex and medullary perfusion. In the present study, we revealed the close relationship between MR_2_* and eGFR. CR_2_* and MR_2_* were decreased and in remission simultaneously in ATIN, demonstrating that inflammation-relating tubular injury was diffusely distributed in the interstitium. The reduction of MR_2_* to CR_2_* ratio detected in ATIN and CTIN declared the involvement of ischemic factors leading to tubular injury. Interestingly, MR_2_* was persistently low against the “pseudo normalization” of CR_2_* with a declining M/CR_2_* ratio, indicated the existence of chronic intrarenal ischemia and hypoxia despite a relatively sufficient oxygen supply than consumption. Intrarenal hypoxia was later emphasized by Sugiyama K et al. that reduced oxygenation but not fibrosis defined by functional MRI predicts the long-term progression of CKD [39]. From the relationship shown in the scatter plot (Fig. 8E), we observed a concurrently lowered MR_2_* with ADC_*b800*_ in ATIN patients [15].

Our study’s limitation was the disability to get direct information on intrarenal perfusion. DW and BOLD imaging were the most frequently used fMR imaging techniques in human diseases. Combining these data and developing new strategies would provide more accurate methods to diagnose, assess, and evaluate the mechanism of diseases [28, 29]. A recent study reported that a combination of renal ADC and parenchymal volume could improve the renal function assessment in CKD [40]. This initial study had limited participants. It is considered more of a proof-of-concept study, limited to have a definite conclusion with these small numbers. One of the difficulties of patient recruitment in this study lies in that multiparameter sequence MR scanning is inevitably time-consuming. Furthermore, image quality was enormously influenced by poor respiratory cooperation, which restricts critically ill AKI patients. As a result, fewer patients were suitable for the study, and some patients enrolled were unable to meet the requirements of the study. To make up for these deficiencies, we tried to follow up with these patients and get comparable fMR parameters in the remission stage of ATIN, hoping to give the possible clinical significance of ADC and R2* values. Even so, it was necessary to recruit more participants to verify our results further.

## Conclusions

This study observed significant reduction and remission of ADC values and R_2_* in the ATIN case series. The ADC_*b800*_ and MR_2_* were concurrently decreased. Thus, further sample recruitment is essential to verify the diagnosis significance of these functional MR parameters and test sensitivity. The pseudo normalization of CR_2_* with persistently lowered MR_2_* in CTIN provided evidence of intrarenal hypoxia.

## Data Availability

These patients were regularly followed up, and the clinical data is traceable. The datasets generated and analyzed during the current study are available from the corresponding author on reasonable request.

## Abbreviations

MR: magnetic resonance
fMR: functional MR
DW: diffuse weighted
BOLD: Blood oxygen level-dependent
ADC: global apparent diffusion coefficient
ATIN: acute tubulointerstitial nephritis
CTIN: chronic tubulointerstitial nephritis
CR_2_*: cortical R_2_* values
MR_2_*: medullary R_2_* values
M/CR_2_*: the ratio of MR_2_* to CR_2_*
